# Variation of functional diversity structure measured as combined species dominance, functional diversity, and functional redundancy in two taxa of ectoparasitic arthropods at two spatial scales: host-associated, ecological, and geographic effects

**DOI:** 10.1017/S0031182024001483

**Published:** 2024-12

**Authors:** Boris R. Krasnov, Michal Stanko, Maxim V. Vinarski, Natalia P. Korallo-Vinarskaya, Irina S. Khokhlova

**Affiliations:** 1 Mitrani Department of Desert Ecology, Swiss Institute for Dryland Environmental and Energy Research, Jacob Blaustein Institutes for Desert Research, Ben-Gurion University of the Negev, Sede Boqer Campus, Midreshet Ben-Gurion, Israel; 2 Institute of Parasitology and Institute of Zoology, Slovak Academy of Sciences, Kosice, Slovakia; 3 Laboratory of Macroecology and Biogeography of Invertebrates, Saint-Petersburg State University, Saint-Petersburg, Russian Federation; 4 Laboratory of Parasitology, Zoological Institute of Russian Academy of Sciences, Saint-Petersburg, Russian Federation; 5 French Associates Institute for Agriculture and Biotechnology of Drylands, Jacob Blaustein Institutes for Desert Research, Ben-Gurion University of the Negev, Sede Boqer Campus, Midreshet Ben-Gurion, Israel

**Keywords:** fleas, gamasid mites, mammals, ternary diagram, traits

## Abstract

The functional diversity structure of a community can be represented as a combination of three additive components (species dominance D, functional redundancy R, and functional diversity Q) (DRQ approach in which different facets of functional differences between species are considered simultaneously). We applied this concept to assemblages of fleas and gamasid mites parasitic on small mammals at continental (across regions of the Palearctic) and regional (across sampling sites in Slovakia) scales and asked: What are the relative effects of host species, biome/habitat type, and geographic locality on the DRQ composition of a parasite assemblage? At the continental scale, regions were partitioned according to predominant biome or geographic position in a continental section. At the regional scale, sampling sites were partitioned according to habitat type or geographic locality. We tested for differences in the functional diversity structure (measured as the DRQ composition) of an ectoparasite assemblage (a) within a host species between biomes/habitat types or continental sections/localities and (b) between host species within a biome/habitat type or a continental section/locality. At both scales, the functional diversity structure of both flea and mite assemblages differed mainly between host species within a biome/habitat or geographic regions/locations, whereas differences in the DRQ composition between biomes/habitats or geographic regions/locations were only detected in a few host species. We compare our results with the results of earlier studies and conclude that the DRQ approach has an advantage over a single diversity metric and allows a better understanding of spatial variation in different facets of ectoparasite diversity.

## Graphical abstract

**Figure:**
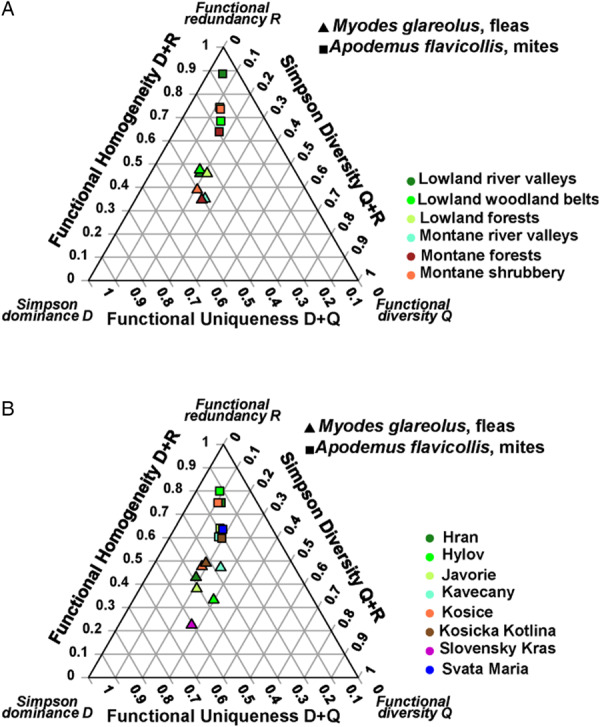

## Introduction

Multiple definitions of biodiversity have been proposed (e.g. Haila and Kouki, 1994; DeLong, 1996; Gaston and Spicer, 2013; Naeem *et al*., 2016). Despite this variety, all of these definitions recognize biodiversity's multifaceted nature. In other words, biodiversity encompasses multiple elements of life's variability, including its compositional, functional, and phylogenetic aspects (e.g. Cadotte *et al*., 2009; Stevens and Gavilanez, 2015). Based on the idea of diversity's multiple facets and the earlier ideas of Rao (1982), Gregorius and Kosman (2017) proposed distinguishing between diversity and dispersion, with the former being related to the number and abundances of observational units (e.g. species) and the latter describing the extent of the difference between these units, arguing that these two types of variation focus on completely different aspects. From this point of view, classical measures of diversity (i.e. taxonomic or compositional diversity) represent diversity *per se*, whereas functional and phylogenetic diversity represent dispersion. Further, we will refer to the latter aspects as ‘diversity’ to be consistent with commonly accepted terminology.

Although different facets of diversity have often been considered in the same study (e.g. Flynn *et al*., 2011, Kamran *et al*., 2011, Krasnov *et al*., 2019, 2023; E-Vojtkó *et al*., 2023), they have been tackled separately rather than simultaneously. Recently, Ricotta *et al*. (2023) proposed a method that summarizes different facets of functional differences between species within sites. In this method, the functional diversity structure is decomposed into three components, namely Rao's functional diversity Q (represented by Rao's quadratic diversity; Rao, 1982), functional redundancy R (de Bello *et al*., 2007), and Simpson dominance (complement of Simpson diversity) D. Rao's functional quadratic diversity Q is an average trait-based (i.e. functional) measure of dissimilarity between two individuals taken randomly with replacement from the site. Functional redundancy R represents the amount of species diversity (i.e. classical Simpson diversity; S) not associated with functional diversity, that is, R = S − Q. Functional redundancy ranges from zero (if all species in a community are maximally dissimilar) to S (if all species in a community are alike). From an ecological perspective, similar species (i.e. those possessing similar traits) in a community are assumed to perform similar functions, so that they are interchangeable, and the loss of a particular species would have a relatively weak effect on community or ecosystem functioning (Naeem, 1998; Petchey *et al*., 2007). Simpson dominance D is the contribution to the mean species similarity obtained if two randomly selected individuals from the same community belong to the same species (Ricotta *et al*., 2023), calculated as D = 1 − S. The three above-described components are thus additive, and their sum equals unity. Ricotta *et al*. (2023) further proposed that these functional diversity components can be used to visualize a community's functional structure on a ternary diagram (called the DRQ ternary diagram). In this case, the ternary diagram is a triangular plot with the vertices representing D, R and Q, and each community being represented by a point whose position is established by the values of the three components. Therefore, the DRQ framework allows comparing functional diversity structure between communities from a more comprehensive perspective than by only using a single diversity measure.

Investigations of species diversity patterns, calculated using Simpson index or its complement, have a long history, beginning from this metric's introduction in 1949 (Simpson, 1949), which was followed by an explosion of studies implementing the Simpson index for a huge variety of taxa, including parasites (Poulin and Morand, 2004). Studies of spatial and/or temporal variation in functional diversity components (functional diversity *per se* and functional redundancy) in a variety of plant and animal taxa began about two decades ago (e.g. Fonseca and Ganade, 2001; Petchey *et al*., 2007; Pillar *et al*., 2013; Violle *et al*., 2014; Biggs *et al*., 2020; Monge-González *et al*., 2021; Lazarina *et al*., 2023). Various patterns have been reported. For example, the functional diversity of freshwater fish communities appeared to be related to habitat quality in terms of land use and riparian vegetation (Stefani *et al*., 2024). Both functional diversity and redundancy were shown to be higher in lowland than in mid-elevation tropical forests (Monge-González *et al*., 2021). The functional diversity and redundancy of parasite communities have been less frequently studied than those of free-living species. Nevertheless, differences in functional diversity and redundancy have been found between host species, habitats, locations, and seasons (Llopis-Belenguer *et al*., 2020; Krasnov *et al*., 2021*a*, 2021*b*), although this was the case for some, but not other, parasite taxa (e.g. Krasnov *et al*., 2021*a*). Importantly, in all the above-mentioned studies, the three components of Ricotta *et al*.'s (2023) framework were considered separately rather than simultaneously, as proposed by the DRQ concept. To the best of our knowledge, the DRQ approach has never been applied to empirical data except for the case study of plant communities in grazed and ungrazed plots of grasslands in Italy carried out by Ricotta *et al*. (2023) to illustrate the use of the concept.

Here, we employed the DRQ approach to study patterns of functional diversity structure in communities of fleas and gamasid mites parasitic on small mammals at two spatial scales, namely (a) a continental scale (across regions from a large part of the central and northern Palearctic) and (b) a regional scale (across sampling sites within a single region; eastern Slovakia). Based on earlier findings on the effects of host species, biome/habitat type, and geographic locality on species dominance, functional diversity, and functional redundancy in parasite communities (see citations above), we asked (a) whether these host-associated, ecological, and geographic effects also hold for functional diversity structure considered as the DRQ composition, and (b) what the relative extent of each of these effects is. At the continental scale, regions were partitioned either by a predominant biome or by their geographic positions (into continental sections), whereas at the regional scale, sampling sites were partitioned either by habitat type or by geographic locality (see Supplementary Material, Appendix 1, Fig. S1 for a conceptual scheme). Consequently, we tested for differences in the functional diversity structure of flea and mite communities (a) within a host species between biomes or continental sections or habitat types or localities and (b) between host species within a biome or a continental section or a habitat type or a locality (see Supplementary Material, Appendix 1, Figs. S2–S3 for conceptual schemes).

## Materials and methods

### Data

At the continental scale, data on the species composition of fleas and gamasid mites (obligatory or facultatively haematophagous species only) were taken from published surveys that reported the number of ectoparasite individuals of a given species collected from a given number of individuals of a given small mammal species (rodents, shrews, moles, and pikas) in 45 (fleas) and 29 (mites) regions of the central and northern Palearctic. The lists of regions, numbers of ectoparasites and host species, maps, respective references, and details on sampling procedures can be found in Krasnov *et al*. (2009, 2015) and Warburton *et al*. (2017).

At the regional scale, data on fleas and haematophagous mites parasitizing small mammals (rodents and shrews) were collected from 1983–2001 from 118 sampling sites distributed across 13 geographic localities in eastern Slovakia (see map in Krasnov *et al*., 2015). Details on sampling procedures and methods for parasitological examination are given elsewhere (Stanko, 1994). The majority of sites were sampled multiple times. For these sites, data of flea and mite counts were pooled for each host species across all sampling periods.

In each region (at the continental scale) or sampling site (at the regional scale), we selected host species in which at least 10 individuals were examined for fleas or mites. Estimation of ectoparasite counts from examination of less than 10 conspecific hosts can be unreliable because of parasite aggregation among host individuals (Poulin, 2007). For the continental scale, this selection resulted in data on 1 727 402 flea and 316 839 mite specimens belonging to 202 and 72 species, respectively, and collected from 541 218 individual mammals belonging to 123 species (for fleas) and 249 313 individual mammals belonging to 70 species (for mites). For the regional scale, the selection resulted in data on 14 670 flea and 60 443 mite specimens belonging to 24 and 20 species, respectively, and collected from 12 700 individual mammals belonging to 12 species (for fleas) and 10 664 individual mammals belonging to 13 species (for mites).

### Flea and mite traits

Both fleas and mites were characterized by five quantitative and either three (fleas) or one (mites) nominal traits. Quantitative traits were: (a) characteristic (mean) abundance on the principal host; (b) the degree of host specificity in terms of the numbers of hosts exploited across a parasite's geographic range; (c) this host spectrum's phylogenetic diversity; (d) body size; and (e) the degree of sexual dimorphism. The rationale and details of the calculations of the mean abundance and the degree of host specificity have been previously described (Poulin *et al*., 2011; Krasnov *et al*., 2013, 2015, 2023; Surkova *et al*., 2018). These variables were controlled for unequal sampling effort (see Krasnov *et al*., 2019). The phylogenetic diversity of a host spectrum was calculated as Faith's (Faith, 1992) phylogenetic diversity. We calculated standardized values of phylogenetic diversity that are independent of host species richness, using the ‘ses.pd’ function of the ‘picante’ package (Kembel *et al*., 2010) implemented in the R Statistical Environment (R Core Team, 2024). Topologies and branch lengths of host phylogenies were taken as 1000-random trees subsets from the 10 000 species-level birth-death tip-dated completed trees for the 5911 mammal species of Upham *et al*. (2019). Then, a consensus tree was constructed using the ‘consensus.edge’ function of the ‘phytools’ package (Revell, 2012), implemented in R and ultrametrized using the ‘force.ultrametric’ function of ‘phytools’. The body size of a flea or a mite species was estimated *via* either maximal body length or the midline length of the dorsal shield, respectively, and taken as the median of the average male and average female body size (see details in Krasnov *et al*., 2013; Surkova *et al*., 2018). The degree of sexual dimorphism was calculated as the logarithmic female-to-male size ratio (Smith, 1999). Nominal trait variables for fleas included (a) microhabitat preference (spending most of their time either on the host's body or in its burrow or nest, or both; see details in Krasnov *et al*., 2015); (b) reproductive season (warm or cold or year-round); and (c) the occurrence and/or number of sclerotized combs (ctenidia) that allow a flea to anchor itself in a host's hair (either no combs or one or two combs). Mite species were characterized by their feeding mode as being either (a) obligatory exclusively haematophageous (feeding solely on the host's blood), (b) obligatory non-exclusively haematophageous (feeding on both the host's blood and small nidicolous arthropods), or (c) facultatively haematophageous (see Radovsky, 1985; Krasnov *et al*., 2013).

### Data partitioning

At the continental scale, we grouped regions according to either predominant biomes (five groups) or their geographic positions (eight continental sections) (Supplementary Material, Appendix 2, Table S1; see also Warburton *et al*., 2017). This was done separately for fleas and mites because of separate databases for the two taxa. At the regional scale, sampling sites were grouped either according to habitat type (10 habitats) or geographic locality (13 localities). Detailed descriptions of habitat types can be found in Krasnov *et al*. (2006*a*) and Gibert *et al*. (2021). This partitioning was carried out for data pooled for fleas and mites because these taxa were sampled in the same places at the same time periods. The majority of sampling sites covered more than one habitat type. These sites were divided into habitat-homogeneous subsites (based on the distribution of trap lines), which were considered separately (in total, 263 subsites).

### Data analyses

Trait data were analysed for fleas and mites separately. First, quantitative trait data were scaled between 0 and 1. Then, we constructed matrices of functional dissimilarities between flea or mites species using the ‘dist.ktab’ function from the R package ‘ade4’ (Thioulouse *et al*., 2018) and calculating Gower's distances (Gower, 1971) between flea or mites species. The latter allows the combination of different types of trait variables (continuous and nominal, in our case) and is unsensitive to missing data. Subsequently, Gower's distances were transformed into Euclidean distances using the ‘lingoes’ function of ‘ade4’.

We calculated the abundance of each flea or mite species for each host species in each region (at the continental scale) or sampling subsite (at the regional scale) as the mean number of individual fleas or mites of a given species recorded on both the infested and uninfested host individuals examined. From these values, the relative abundances of fleas or mites per host species per region or sampling subsite were then calculated. These data, combined with the matrices of functional distances, were used to calculate Rao's functional diversity Q of the flea and mite assemblages of a given host in a given region or sampling subsite using the ‘QE’ function of the R package ‘adiv’ (Pavoine, 2020). Simpson diversity S was calculated using the ‘speciesdiv’ function of the ‘adiv’ package with option method = ‘GiniSimpson’. Then, Simpson's dominance D and functional redundancy R were calculated as D = 1 − S and R = S − Q, respectively (see above).

For the continental scale, we tested for differences in the functional diversity structure (represented as the combination of D, R, and Q values; further referred to as the DRQ composition) (a) within a host species between biomes or continental sections and (b) between host species within a biome or a continental section (see conceptual scheme in Supplementary Material, Appendix 1, Fig. S2). For the regional scale, the differences in the functional diversity structure were tested (a) within a host species between habitats or localities and (b) between host species within a habitat or a locality (see conceptual scheme in Supplementary Material, Appendix 1, Fig. S3). The tests were carried out using a distance-based multivariate analysis of variance (db_MANOVA) implemented in the R package ‘PERMANOVA’ (Vicente-Gonzalez and Vicente-Villardon, 2021). This is a non-parametric multivariate generalization of a classical ANOVA, used for testing the differences between two or more groups of plots based on every possible dissimilarity measure by comparing the within-group *vs* the between-group dissimilarities (Anderson, 2001). Pairwise dissimilarities between host species, biomes, continental sections, habitats, or localities (see above), in the functional diversity structure of flea or mite assemblages (the DRQ components), were calculated using the Bray-Curtis dissimilarity. Then, *P* values were obtained by 10 000 random permutations of flea or mite assemblages between respective host species, biomes, continental sections, habitats, or localities. Prior to the within-host species analyses, we selected those host species (a) that occurred in at least two biomes, continental sections, habitat types, or localities and (b) in which parasite assemblages were represented by at least three samples. Prior to the between-host species analyses, we selected those biomes, continental sections, habitats, or localities in which parasite assemblages of at least two host species were represented by at least three samples.

As mentioned above, Ricotta *et al*. (2023) proposed visualizing a community's functional structure on the DRQ ternary diagram, which is a triangular plot with the vertices being *D*, *R*, and *Q*. The corners of the triangle correspond to the value of a given component being 1, while the values of the remaining components are zeros. The component's values decrease linearly with increasing distance from the respective corner. Three facets of functional diversity are thus manifested by a contrast between a vertex and its opposing edge, when the two components are added and the third is considered as a contrast. In particular, R + Q represents Simpson diversity, D + R represents functional homogeneity, and D + Q represents functional uniqueness (see Ricotta *et al*., 2023 for details). We generated the DRQ diagrams using the R package ‘Ternary’ (Smith, 2017).

Finally, to further test for significant differences in each single diversity measure (D, R, and Q) (a) within a host species between biomes, continental sections, habitats, or localities and (b) between host species within a biome, a continental section, a habitat, or a locality and following Ricotta *et al*. (2023), we applied univariate ANOVAs with 10 000 random permutations of parasite assemblages between the respective groups. This was done using the R package ‘RRPP’ (Collyer and Adams, 2018).

## Results

### The continental scale

**Figure 1.**
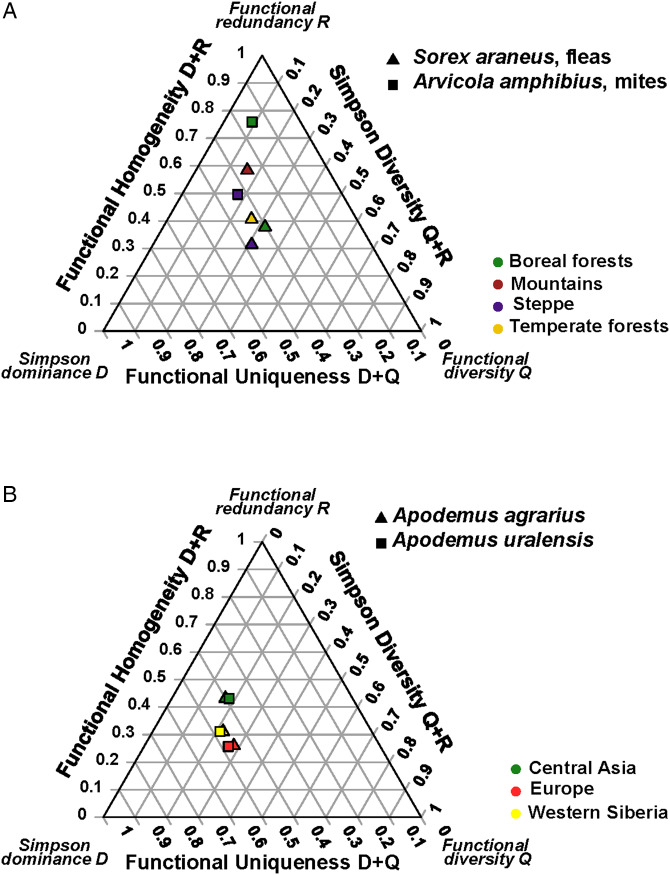
The DRQ ternary diagrams for (A) flea assemblages of *Sorex minutus* and mite assemblages of *Arvicola amphibius* in different biomes and (B) flea assemblages of *Apodemus agrarius* and *Apodemus uralensis* in different continental sections.

Differences in the DRQ composition of parasite assemblages between biomes were found only in the flea assemblages of *Sorex minutus* and the mite assemblages of *Arvicola amphibius* (*P* < 0.05 for both) (see detailed results for all host species in Supplementary Material, Appendix 2, Table S3). Univariate ANOVAs on separate D, R, and Q components in these assemblages demonstrated significant biome effects on the values of (a) D and R (but not Q) in fleas and (b) all three components in mites (Fig. 1A) (Supplementary Material, Appendix 2, Table S4). Between continental sections, the DRQ composition of assemblages harboured by the same host species differed significantly in the fleas of *Apodemus agrarius* and *Apodemus uralensis* (*P* < 0.05 for both; Fig. 1B) but not in the mites of any host (see detailed results for all host species in Supplementary Material, Appendix 2, Table S5). In the fleas of both *Apodemus* hosts, significant differences between continental sections were found in the D and R (but not Q) values (Supplementary Material, Appendix 2, Table S6).

**Figure 2.**
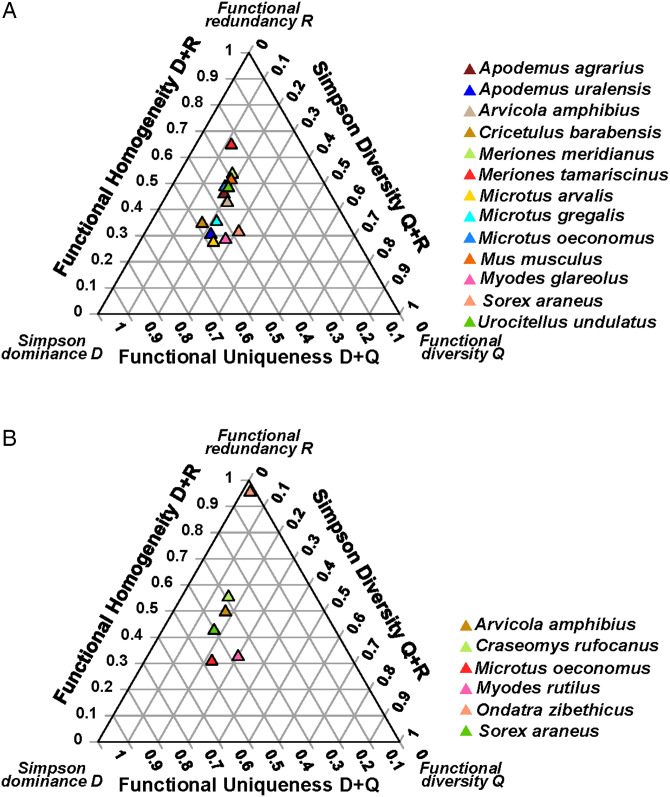
The DRQ ternary diagrams for (A) flea and (B) mite assemblages of different host species in the steppe biome.

Host-associated effects on the DRQ composition of flea and mite assemblages were found in four of five biomes (except deserts) for fleas and two of three biomes (except temperate forests) for mites (Table 1). Ectoparasite assemblages differed between species in the values of all three DRQ components, except R in fleas in mountain and steppe biomes (Supplementary Material, Appendix 2, Table S7). Illustrative ternary diagrams for flea and mite assemblages in steppes are presented in Fig. 2.

**Table 1.** Summary of distance-based multivariate analyses of variance (db-MANOVAs) testing for differences in the functional structure of flea and mite communities (the DRQ composition; see text for explanations) between host species within a biome at the continental scale

Parasite	Biome	Nsp	Proportion of variance explained	*F*	*P*
Fleas	Boreal forests	12	0.56	4.87	<0.001
	Deserts	6	0.11	0.39	0.87
	Mountains	15	0.38	2.31	0.01
	Steppes	13	0.41	2.04	0.04
	Temperate forests	16	0.64	5.66	<0.001
Mites	Boreal forests	19	0.52	4.76	<0.001
	Steppes	6	0.64	5.25	0.01
	Temperate forests	6	0.19	0.69	0.02

Nsp, number of host species for which flea or mite DRQ composition was calculated.

**Figure 3.**
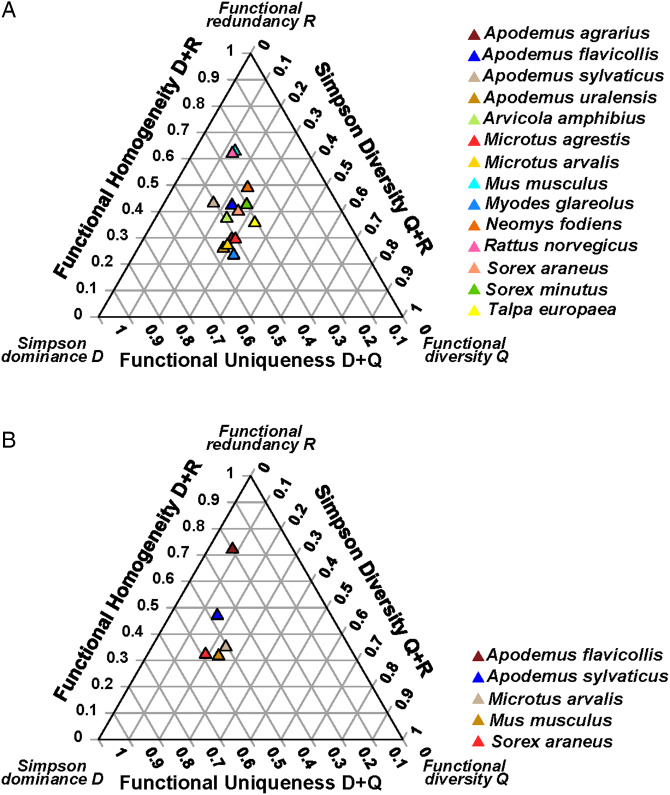
The DRQ ternary diagrams for (A) flea and (B) mite assemblages of different host species in Europe.

In four of six (except central Asia and southern Siberia) continental sections, the DRQ compositions differed significantly between flea assemblages harboured by different host species (Table 2). In mite assemblages, significant differences in the DRQ composition were detected in only two of five continental sections (Europe and western Siberia; Table 2). In all continental sections, a significant effect of host species identity was found on the values of all three separate DRQ components, except D values in western Siberia and R values in the Caucasus (Supplementary Material, Appendix 2, Table S8; see illustrative ternary diagram for Europe in Fig. 3).

**Table 2.** Summary of distance-based multivariate analyses of variance (db-MANOVAs) testing for differences in the functional structure of flea and mite communities (the DRQ composition; see text for explanations) between host species within a continental section at the continental scale

Parasite	Continental section	Nsp	Proportion of variance explained	*F*	*P*
Fleas	Asian Far East	6	0.79	12.17	<0.001
	Caucasus	6	0.48	2.79	0.04
	Central Asia	20	0.27	1.48	0.12
	Europe	14	0.40	2.81	0.01
	Southern Siberia	4	0.20	0.65	0.59
	Western Siberia	14	0.40	1.86	0.04
Mites	Asian Far East	2	0.10	0.42	0.93
	Europe	5	0.47	3.61	0.02
	Northern Siberia	3	0.07	0.35	0.85
	Southern Siberia	3	0.28	1.55	0.19
	Western Siberia	14	0.60	5.78	<0.001

Nsp, number of host species for which flea or mite DRQ composition was calculated.

### The regional scale

**Figure 4.**
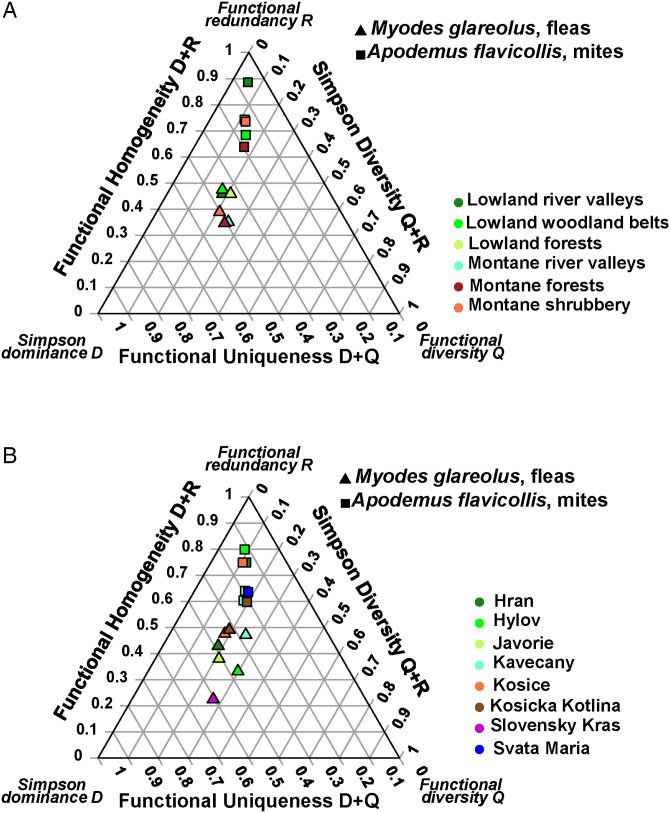
The DRQ ternary diagrams for flea and mite assemblages of *Myodes glareolus* and *Apodemus flavicollis*, respectively, in different (A) habitat types and (B) localities across Slovakia.

Effects of habitat type on the DRQ composition were found in the flea assemblages of *M. glareolus* and the mite assemblages of *A. flavicollis* (Table 3; Fig. 4A). These differences were accompanied by those in the separate DRQ components, except the values of Q in *M. glareolus* (Supplementary Material, Appendix 2, Table S9; Fig. 4A). Differences in the DRQ composition between geographic localities were detected for fleas of three *Apodemus* hosts (*A. agrarius*, *A. flavicollis,* and *A. uralensis)* and *M. glareolus*, whereas for mites, this was the case for *A. flavicollis* only (Table 4; Fig. 4B). Separate analyses of D, R, and Q components proved significant between-locality differences in the three components' values, except R in *A. agrarius* and Q in *A. uralensis* (Supplementary Material, Appendix 2, Table S10).

**Table 3.** Summary of distance-based multivariate analyses of variance (db-MANOVAs) testing for differences in the DRQ composition of flea or mite assemblages harboured by the same host species between habitat types at the regional scale

Parasite	Host species	Np	Proportion of variance explained	*F*	*P*
Fleas	*Apodemus agrarius*	80	0.06	1.01	0.43
	*Apodemus flavicollis*	108	0.09	1.92	0.09
	*Apodemus uralensis*	35	0.05	0.59	0.69
	*Microtus arvalis*	17	0.24	2.23	0.16
	*Myodes glareolus*	79	0.21	3.80	<0.001
Mites	*Apodemus agrarius*	70	0.04	0.54	0.78
	*Apodemus flavicollis*	107	0.12	2.61	0.03
	*Apodemus uralensis*	28	0.04	0.56	0.57
	*Microtus arvalis*	22	0.03	0.17	0.94
	*Microtus subterraneus*	17	0.07	0.54	0.62
	*Myodes glareolus*	23	0.23	1.85	0.17

Np, number of populations of a host species for which flea or mite DRQ composition was calculated.

**Table 4.** Summary of distance-based multivariate analyses of variance (db-MANOVAs) testing for differences in the DRQ composition of flea or mite assemblages harboured by the same host species between localities at the regional scale

Parasite	Host species	Np	Proportion of variance explained	*F*	*P*
Fleas	*Apodemus agrarius*	80	0.14	2.35	0.04
	*Apodemus flavicollis*	107	0.25	4.81	<0.001
	*Apodemus uralensis*	36	0.15	2.81	0.04
	*Microtus arvalis*	16	0.00	0.03	0.89
	*Myodes glareolus*	70	0.31	4.83	<0.001
Mites	*Apodemus agrarius*	68	0.06	1.03	0.38
	*Apodemus flavicollis*	103	0.14	2.54	0.02
	*Apodemus uralensis*	29	0.14	2.20	0.10
	*Microtus arvalis*	24	0.10	0.72	0.56
	*Microtus subterraneus*	11	0.03	0.12	0.93
	*Myodes glareolus*	20	0.09	0.52	0.71

Np, number of populations of a host species for which flea or mite DRQ composition was calculated for at least two populations within a locality.

**Figure 5.**
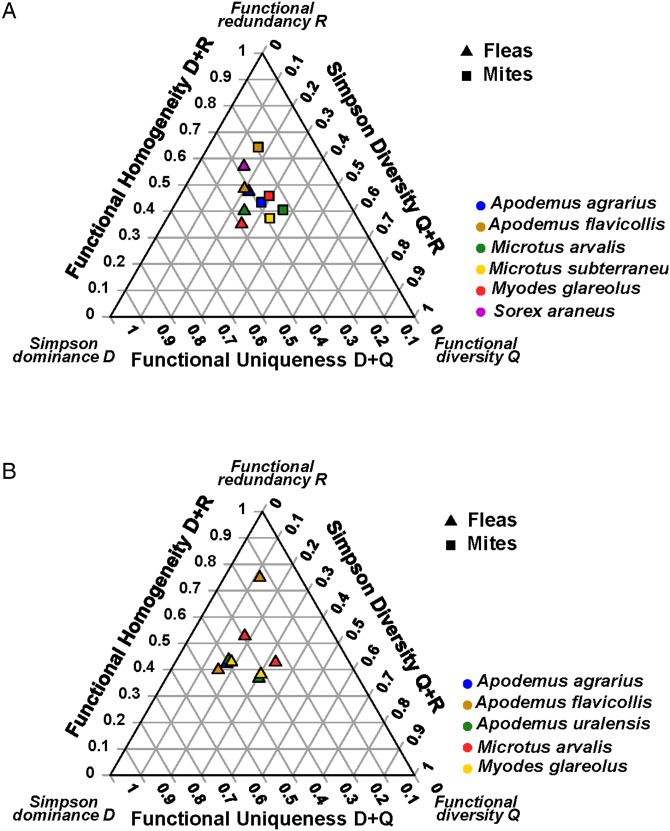
The DRQ ternary diagrams for flea assemblages and mite assemblages in different host species in (A) in montane river valleys and (B) the vicinity of the Hran village in Slovakia.

Host-associated effects on the DRQ composition were revealed in three of seven and five of six habitat types for flea and mite assemblages, respectively (Table 5; see illustrative ternary diagram for montane river valleys in Fig. 5A). Univariate ANOVAs demonstrated significant differences in the values of the separate DRQ components of these assemblages, except Q for both fleas and mites in montane habitats and R in fleas from lowland fields (Supplementary Material, Appendix 2, Table S11).

**Table 5.** Summary of distance-based multivariate analyses of variance (db-MANOVAs) testing for differences in the DRQ composition between flea or mite assemblages of different host species within a habitat type at the regional scale

Parasite	Habitat type	Nsp	Proportion of variance explained	*F*	*P*
Fleas	Lowland fields	3	0.19	4.78	0.01
	Lowland forests	3	0.04	0.55	0.64
	Lowland river valleys	4	0.09	0.88	0.45
	Lowland woodland belts	5	0.08	1.85	0.10
	Montane forests	3	0.12	2.62	0.07
	Montane river valleys	5	0.15	2.99	0.02
	Montane shrubbery	2	0.29	5.38	0.02
Mites	Lowland fields	3	0.11	1.68	0.19
	Lowland forests	2	0.52	23.98	<0.001
	Lowland river valleys	5	0.51	8.57	<0.001
	Lowland woodland belts	7	0.36	6.51	<0.001
	Montane river valleys	5	0.27	4.75	<0.001
	Montane shrubbery	3	0.20	3.89	0.02

Np, number of populations of a host species for which flea or mite DRQ composition was calculated.

Within a locality, the DRQ composition differed significantly between hosts in four of eight localities for fleas and five of seven localities for mites (Table 6; see illustrative ternary diagram for the vicinity of the village of Hran in Fig. 5B). In these localities, the host-associated effect on the values of separate DRQ components was significant, except R in two localities for fleas and Q in one locality for mites (Supplementary Material, Appendix 2, Table S12).

**Table 6.** Summary of distance-based multivariate analyses of variance (db-MANOVAs) testing for differences in the DRQ composition between flea or mite assemblages of different host species within a locality at the regional scale

Parasite	Locality	Nsp	Proportion of variance explained	*F*	*P*
Fleas	Hran	5	0.09	2.87	0.01
	Hylov	3	0.32	5.05	0.01
	Javorie	4	0.19	3.84	0.01
	Kavecany	3	0.20	1.90	0.16
	Kosice	3	0.11	2.01	0.13
	Kosicka Kotlina	4	0.32	4.15	<0.001
	Slovensky Kras	2	0.86	25.40	0.06
	Svata Maria	4	0.20	1.95	0.11
Mites	Hran	5	0.48	22.27	<0.001
	Hylov	2	0.65	26.51	<0.001
	Javorie	5	0.30	4.07	<0.001
	Kavecany	3	0.14	0.88	0.45
	Kosice	5	0.41	5.10	0.00
	Kosicka Kotlina	5	0.15	1.09	0.37
	Svata Maria	3	0.28	4.38	0.02

Np, number of populations of a host species for which flea or mite DRQ composition was calculated.

## Discussion

We found that, in general, the variation in the DRQ composition could have been affected by host-associated (host species identity), ecological (biomes of habitats), and geographic (continental sections or localities) factors. The effect of host species identity on functional diversity structure, measured as the DRQ composition, was substantially stronger than that of ecological or geographic factors. In fact, 47 tests in our study involving between-host comparisons produced 29 significant results (ca. 62%), whereas 65 tests involving within-host comparisons produced only 11 significant results (ca. 17%).

### Between-host variation in functional diversity structure

The most straightforward explanation of the effect of host species on the DRQ composition is the between-host differences in their flea and/or mite species compositions, traits, and relative abundances. This is because each host species possesses certain traits that determine the ability of a given ectoparasite species (also possessing certain traits) to exploit this host successfully. In other words, there is a complementarity between host and parasite traits (McQuaid and Britton, 2013). For example, a correlation between the diameter of hair-shafts in pocket gophers and the width of head grooves of chewing lice (that allows them to grasp the hair of the host) results in the ability of louse species with a certain width of the head-groove to exploit only hosts with the complementary hair-shaft diameter (Morand *et al*., 2000). In fleas, species with well-developed ctenidia mainly parasitize small-bodied hosts (Krasnov *et al*., 2016). The ctenidia enhance flea attachment to the host hair, thus resisting dislodgement by host grooming (Humphries, 1967). Self-grooming is more vigorous in smaller hosts that cannot tolerate high numbers of ectoparasites per unit body surface (Mooring *et al*., 2000). The reason behind the differences in the relative abundances of fleas and mites between hosts is an interplay between species-specific parasite abundance levels (Krasnov *et al*., 2006*b*; Korallo-Vinarskaya *et al*., 2009) and between-host variation in the abundance of the same parasite species (Poulin, 2005). Furthermore, 17 of 29 parasite assemblages, in which significant between-host differences were detected, demonstrated this significance in all three separate DRQ components. In the remaining 12 assemblages, Simpson dominance D, functional redundancy R and functional diversity Q did not differ between host species in one, six and five assemblages, respectively. This suggests that the lack of between-host variation in R or Q in some assemblages does not strongly affect general variation in functional diversity structure, whereas D contributes most to the DRQ variation.

Significant differences between host species within a biome, a continental section, a habitat, or a locality does not, however, mean that all species differed from one another in the DRQ composition. This merely indicates the occurrence of the DRQ differences in the majority of, but not all, species pairs (compare, for example, points *A. agrarius* and *Microtus arvalis* for *vs* points for *A. agrarius* and *Microtus oeconomus* in Fig. 2A), suggesting overlaps in the parasite community composition (including relative abundances) of some host species. The between-host overlap in flea or mite community composition can be caused by the relatively low degree of host specificity in both taxa (Krasnov, 2008 for fleas; Dowling, 2006 for mites). Another reason for this is that both fleas and mites are nidicolous and spend much of their (or even their entire) lives in hosts' burrows. Small mammals often visit each other's burrows where between-host ectoparasite exchange often takes place (e.g. Friggens *et al*., 2010).

Nevertheless, in some biomes, continental sections, habitats, and localities, no significant between-host differences in the DRQ composition were found. This can be associated with environmental conditions affecting (a) the microclimate (air temperature and relative humidity) in hosts' burrows and (b) the patterns of small mammals' spatial distribution and behaviour. The effect of burrow microclimate on fleas and mites is well known (Marshall, 1981; Radovsky, 1985; Krasnov, 2008), with both taxa being sensitive to extremely low and extremely high air temperatures and relative humidities. Therefore, harsh environmental conditions may affect parasite distribution, thus smoothing over the differences between host species due to the selective survival of parasites that can tolerate these conditions. A harsh environment may also affect host density, which, in turn, may affect the probability of ectoparasite exchange. This can be the reason for the lack of between-host differences in the DRQ composition of flea assemblages in deserts and mite assemblages in northern Siberia, although it is difficult to propose reasons for other biomes, continental sections, habitats, and localities. We recognize, however, that this explanation is highly speculative and warrants further investigation.

### Within-host variation in functional diversity structure

The general lack of significant differences in the DRQ composition of the same host across ecological (biomes, habitats) or geographic (continental sections, localities) units suggests the relative stability of functional diversity structure in a host species. One of the reasons for this may be low spatial variation in those traits of a host species that determine the complementary traits of flea or mite species enabling them to exploit this host. This could be a mechanism behind lower variation of flea and mite species diversity (at least in terms of species richness) between populations of the same host than between host species (Krasnov *et al*., 2005, 2008). However, our results regarding weak (if any) spatial variation in the DRQ composition seem to contradict earlier results on the pattern of distance-decay community similarity (a decrease in community similarity with an increase in spatial distance) (Nekola and White, 1999; Poulin, 2003; Krasnov *et al*., 2005 for fleas; but see Korallo-Vinarskaya *et al*., 2009 for mites) and on spatial factors' effect on the functional beta-diversity of flea and mite communities (Krasnov *et al*., 2019).

Nevertheless, the DRQ composition of the flea and/or mite assemblages of 11 hosts varied significantly across biomes, continental sections, habitats, and localities. In half (five) of these mammals, functional diversity Q did not differ between ecological or geographic units. Therefore, Simpson's dominance D and functional redundancy R contributed most to spatial variation in these hosts' functional diversity structure, suggesting that the functional similarity between two randomly selected individuals of different species was spatially invariant. One of the explanations for spatial variation in functional diversity structure could be variation in parasites' species composition, traits, and relative abundances in hosts with larger geographic ranges (Krasnov *et al*., 2004; Shenbrot *et al*., 2007; Leiva *et al*., 2020). However, this explanation is unlikely to support our results because the significant differences in the DRQ composition of a host species did not depend on the number of biomes/habitats or continental sections/localities that a host occupied. For example, significant between-biome differences in the DRQ composition at the continental scale were found in the fleas of *S. araneus* that occurred in three biomes but not in *Mus musculus* that occurred in five biomes. Similarly, *A. agrarius* and *A. flavicollis* in Slovakia each occupied six habitat types, but significant between-habitat differences in the DRQ composition were detected in the latter and not in the former. In other words, the probability of ecological and geographic effects on the DRQ composition of a host species' flea and mite assemblages were not associated with its ecological tolerance and geographic range.

Spatial intraspecific variation in the DRQ composition of flea or mite assemblages in some host species may result from a spatial variation in host traits that affects parasite communities. For example, the basal metabolic rate (BMR) correlates positively with parasite species richness because hosts exposed to diverse immunological challenges should invest in a high BMR to compensate for a costly immune response (Morand and Harvey, 2000; but see Krasnov *et al*., 2004). It has been shown that the BMR could vary between different populations of the same species (Novoa *et al*., 2005; Bozinovic *et al*., 2009; Tieleman *et al*., 2009). Burrow structure is another trait that may vary between habitats or localities, thus possibly affecting variation in species (and, consequently, trait) composition and the relative abundances of nidicolous ectoparasites such as fleas and mites in small mammals (Shenbrot *et al*., 2002). Burrow structure variation results from between-habitat differences in soil and vegetation structure, as well as climatic conditions. These differences lead to differences in burrow microclimate and, ultimately, ectoparasite communities (Krasnov *et al*., 1998). It remains unclear, however, why the above-mentioned traits may vary spatially in some but not other host species.

In general, a substantially stronger effect of host-associated than ecological or geographic factors on the functional diversity structure was characteristic for both fleas and mites. Earlier studies that considered the effects of environmental, host-associated, and spatial factors on separate measures of the species diversity, functional diversity, and/or functional redundancy metrics of these two taxa produced different results. For example, Krasnov *et al*. (2005) reported a distance-decay of similarity in the species composition of flea assemblages in six rodent species, while in five of them (except *A. uralensis*), we did not find spatial variation in the DRQ composition. Although the species diversity of both fleas and mites, as well as the functional diversity of mites, have been shown to be mainly affected by host-associated factors (as in this study), flea functional diversity appeared to be equally affected by host-associated and ecological factors (unlike in this study) (Krasnov *et al*., 2019). The functional redundancy (measured as functional uniqueness) of flea assemblages in the African rodents *Rhabdomys pumilio* and *Rhabdomys dilectus* differed between these hosts (as in this study), as well as between biomes (unlike in this study), although the latter was true for *R. pumilio* only (Krasnov *et al*., 2021*a*). We thus conclude that the DRQ framework allows a better understanding of the patterns of variation in different facets of ectoparasite diversity as compared with analysing different diversity indices separately.

## Supporting information

Krasnov et al. supplementary material Krasnov et al. supplementary material

## Data Availability

Raw data can be obtained from the corresponding author upon request.

## Supplementary material

The supplementary material for this article can be found at https://doi.org/10.1017/S0031182024001483
