# Supplementary material for: Variation of functional diversity structure measured as combined species dominance, functional diversity, and functional redundancy in two taxa of ectoparasitic arthropods at two spatial scales: host-associated, ecological, and geographic effects
Source: Parasitology. 2024 Dec 12;151(14):1570–9. doi: 10.1017/S0031182024001483 (PMC12052427; doi:10.1017/S0031182024001483)
Supplement: Krasnov et al. supplementary material [file S0031182024001483sup001.docx]

**Appendix 1. Supplementary Figures**

**Fig. S1.** Conceptual scheme of the partitioning of data at the continental and regional scales.


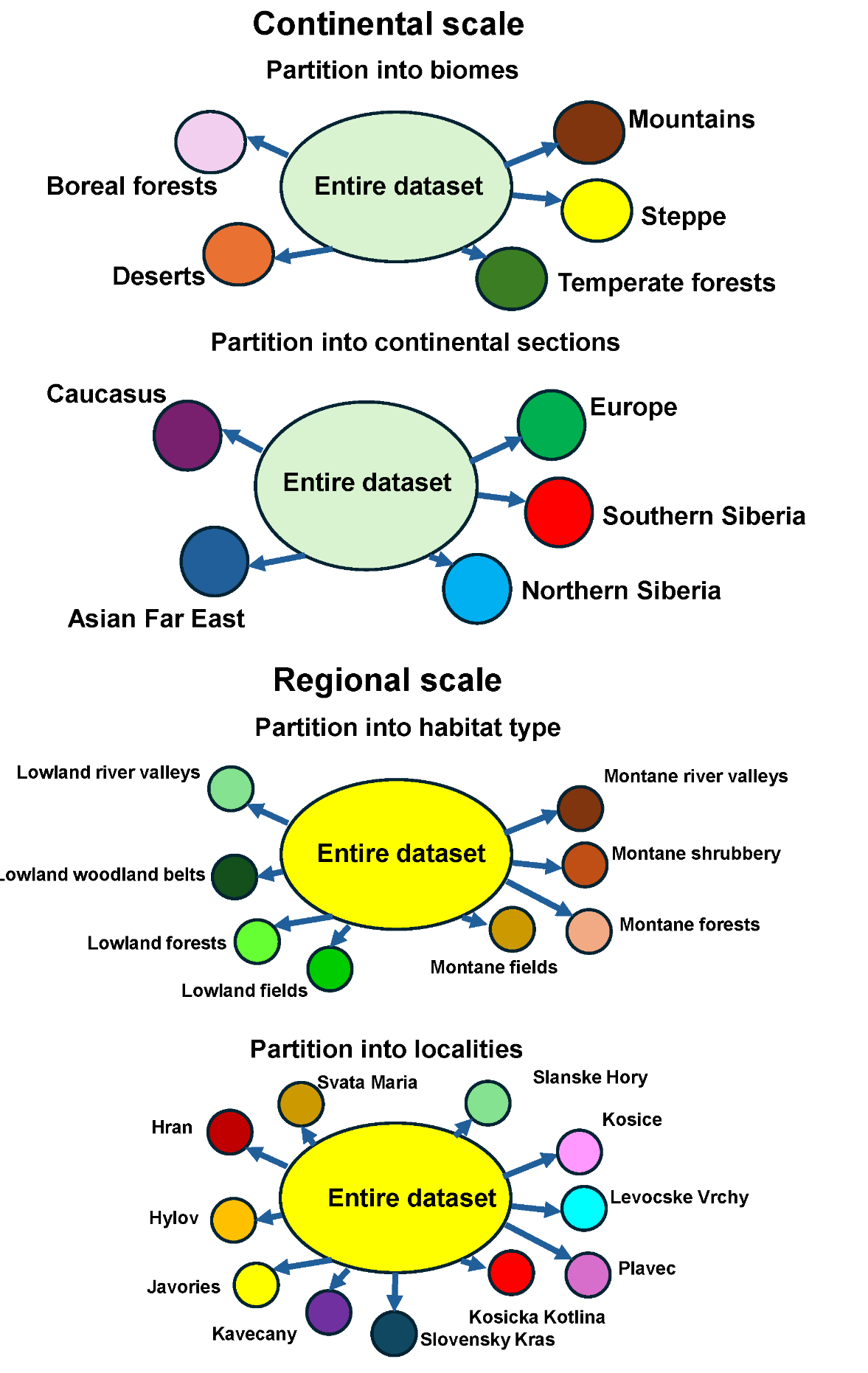


**Fig. S2.** Conceptual scheme of data analyses at the continental scale. Arrows denote comparisons.


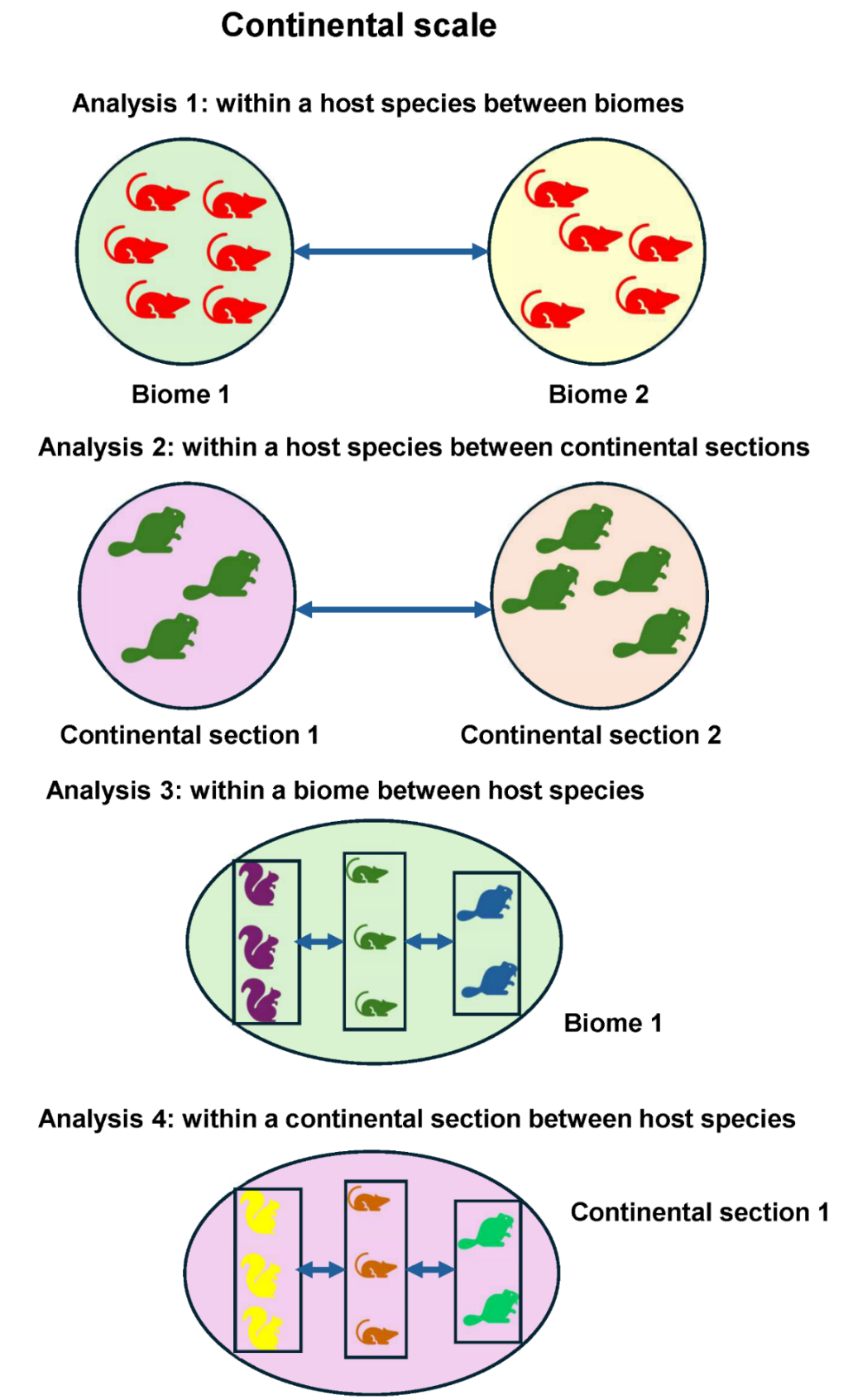


**Fig. S3.** Conceptual scheme of data analyses at the regional scale. Arrows denote comparisons.


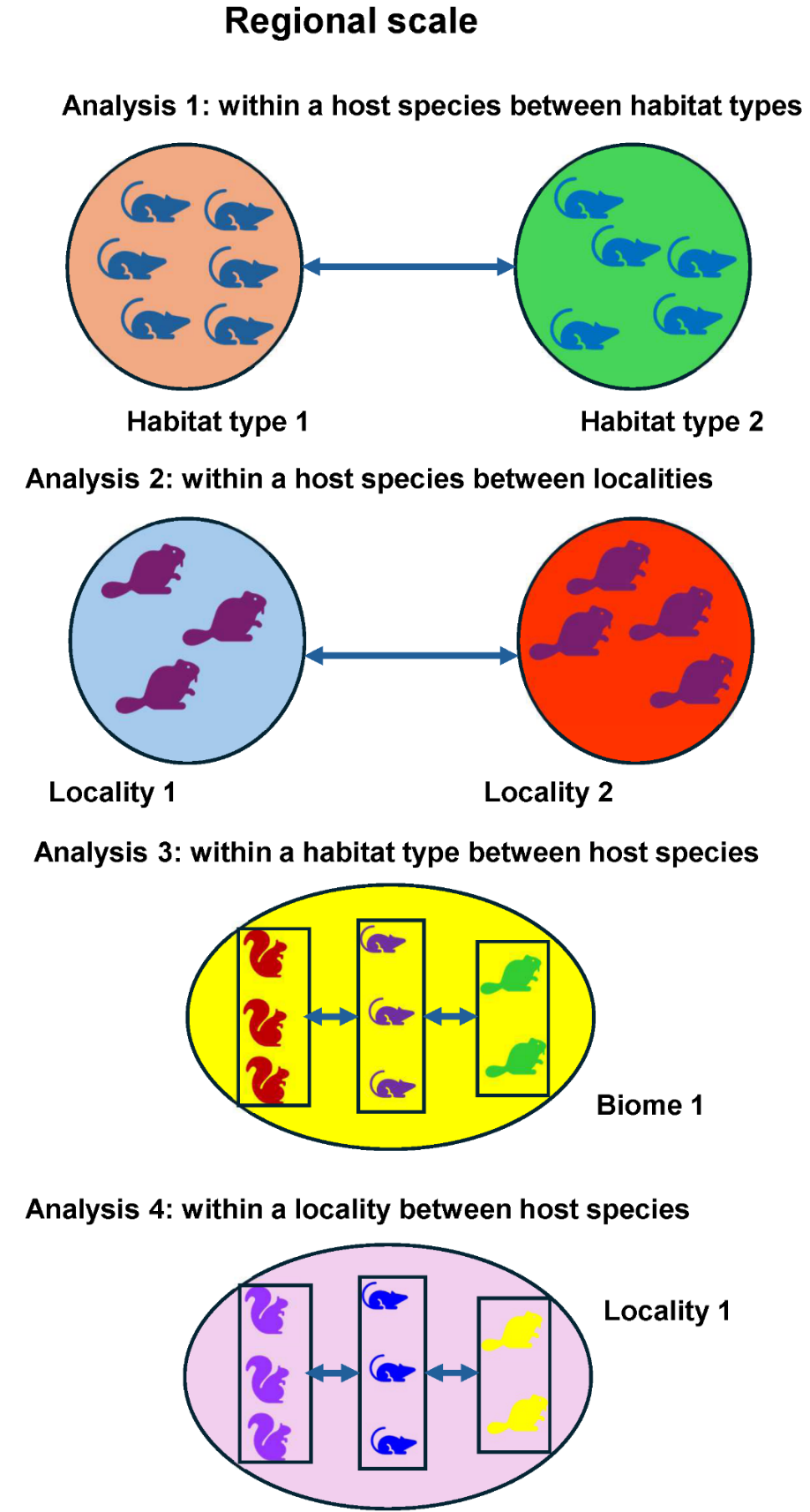


**Appendix 2. Supplementary Tables**

**Table S1.** Partitioning of data at the continental scale (separately for fleas and mites; see the main text for explanation) according to predominant biome (=biomes) or geographic position (=continental sections).

| Partitioning | Biome | Continental section | Number of regions | |
| --- | --- | --- | --- | --- |
|  |  |  | For fleas | For mites |
| Predominant biome | Boreal forests |  | 9 | 15 |
|  | Deserts |  | 5 |  |
|  | Mountains |  | 13 |  |
|  | Steppes |  | 11 | 9 |
|  | Temperate forests |  | 7 | 5 |
| Geographic position |  | Asian Far East | 6 | 2 |
|  |  | Caucasus | 5 |  |
|  |  | Central Asia | 14 |  |
|  |  | Europe | 7 | 8 |
|  |  | Eastern Siberia |  | 2 |
|  |  | Northern Siberia | 2 | 5 |
|  |  | Southern Siberia | 6 | 4 |
|  |  | Western Siberia | 5 | 8 |

**Table S2.** Partitioning of data at the regional scale (pooled for fleas and mites; see the main text for explanation) according to predominant habitat type (=habitat) or geographic position (=locality). The majority of sampling sites covered more than one habitat type. These sites were divided into subsites that are homogeneous from a habitat perspective (based on distribution of trap lines), which were then considered separately.

| Partitioning | Habitat | Locality | Number of sampling subsites |
| --- | --- | --- | --- |
| Habitat type | Lowland river valleys |  | 36 |
|  | Lowland woodland belts |  | 59 |
|  | Lowland fields |  | 33 |
|  | Lowland forests |  | 40 |
|  | Lowland shrubbery |  | 5 |
|  | Montane river valleys |  | 36 |
|  | Montane woodland belts |  | 9 |
|  | Montane fields |  | 9 |
|  | Montane forests |  | 27 |
|  | Montane shrubbery |  | 9 |
| Geographic position |  | Kyjov | 1 |
|  |  | Hran | 106 |
|  |  | Hylov | 8 |
|  |  | Javorie | 31 |
|  |  | Kavecany | 11 |
|  |  | Kosice | 30 |
|  |  | Kosicka Kotlina | 34 |
|  |  | Levocske Vrchy | 34 |
|  |  | Plavec | 2 |
|  |  | Slanske Vrchy | 2 |
|  |  | Slovensky Kras | 4 |
|  |  | Stos | 1 |
|  |  | Svata Maria | 31 |

**Table S3.** Continental scale. Summary of distance-based multivariate analyses of variance (db-MANOVAs) testing for differences in the DRQ composition in flea or mite assemblages harboured by the same host species in different biomes. Np: number of populations of a host species for which flea or mite DRQ composition was calculated.

| Parasite | Host species | Np | Proportion of variance explained | *F* | *p* |
| --- | --- | --- | --- | --- | --- |
| Fleas | *Apodemus agrarius* | 17 | 0.32 | 2.00 | 0.14 |
|  | *Apodemus uralensis* | 15 | 0.22 | 1.72 | 0.22 |
|  | *Arvicola amphibius* | 12 | 0.27 | 1.68 | 0.21 |
|  | *Cricetulus migratorius* | 14 | 0.16 | 2.23 | 0.15 |
|  | *Meriones meridianus* | 6 | 0.14 | 0.63 | 0.59 |
|  | *Microtus agrestis* | 8 | 0.28 | 2.38 | 0.14 |
|  | *Microtus arvalis* | 17 | 0.11 | 0.87 | 0.45 |
|  | *Microtus oeconomus* | 9 | 0.44 | 5.50 | 0.06 |
|  | *Mus musculus* | 27 | 0.23 | 1.68 | 0.17 |
|  | *Myodes glareolus* | 12 | 0.19 | 1.07 | 0.36 |
|  | *Myodes rutilus* | 11 | 0.37 | 5.28 | 0.06 |
|  | *Neomys fodiens* | 9 | 0.52 | 3.30 | 0.10 |
|  | *Sorex araneus* | 16 | 0.39 | 3.04 | 0.04 |
| Mites | *Apodemus agrarius* | 6 | 0.24 | 1.24 | 0.25 |
|  | *Arvicola amphibius* | 9 | 0.77 | 10.03 | 0.03 |
|  | *Craseomys rufocanus* | 11 | 0.39 | 2.52 | 0.12 |
|  | *Microtus arvalis* | 7 | 0.01 | 0.03 | 0.90 |
|  | *Microtus oeconomus* | 17 | 0.32 | 3.28 | 0.06 |
|  | *Mus musculus* | 6 | 0.26 | 1.37 | 0.34 |
|  | *Myodes glareolus* | 11 | 0.02 | 0.21 | 0.80 |
|  | *Myodes rutilus* | 18 | 0.10 | 0.86 | 0.48 |
|  | *Ondatra zibethicus* | 7 | 0.18 | 1.07 | 0.43 |
|  | *Sorex araneus* | 17 | 0.07 | 0.55 | 0.57 |

**Table S4.** Continental scale. Summary of permutational univariate ANOVAs testing for differences in the DRQ values (D: Simpson’s dominance; Q: Rao’s quadratic diversity; R: functional redundancy) of flea and mite assemblages harboured by the same host species in different biomes.

| Parasite | Host species | D/R/Q | *R^2^* | *F* | *p* |
| --- | --- | --- | --- | --- | --- |
| Fleas | *Apodemus agrarius* | D | 0.35 | 2.30 | 0.10 |
|  |  | R | 0.27 | 1.63 | 0.19 |
|  |  | Q | 0.28 | 1.65 | 0.22 |
|  | *Apodemus uralensis* | D | 0.20 | 1.47 | 0.26 |
|  |  | R | 0.55 | 7.32 | 0.01 |
|  |  | Q | 0.09 | 0.62 | 0.54 |
|  | *Arvicola amphibius* | D | 0.29 | 1.84 | 0.21 |
|  |  | R | 0.31 | 2.03 | 0.18 |
|  |  | Q | 0.13 | 0.65 | 0.54 |
|  | *Cricetulus migratorius* | D | 0.16 | 2.35 | 0.14 |
|  |  | R | 0.20 | 2.91 | 0.11 |
|  |  | Q | 0.11 | 1.49 | 0.27 |
|  | *Meriones meridianus* | D | 0.13 | 0.59 | 0.52 |
|  |  | R | 0.01 | 0.05 | 0.85 |
|  |  | Q | 0.24 | 1.25 | 0.25 |
|  | *Microtus arvalis* | D | 0.29 | 2.39 | 0.17 |
|  |  | R | 0.35 | 3.16 | 0.08 |
|  |  | Q | 0.23 | 1.84 | 0.18 |
|  | *Microtus oeconomus* | D | 0.10 | 0.75 | 0.49 |
|  |  | R | 0.29 | 2.83 | 0.09 |
|  |  | Q | 0.08 | 0.58 | 0.57 |
|  | *Mus musculus* | D | 0.44 | 5.52 | 0.07 |
|  |  | R | 0.65 | 12.94 | 0.02 |
|  |  | Q | 0.30 | 3.02 | 0.14 |
|  | *Myodes glareolus* | D | 0.24 | 1.73 | 0.17 |
|  |  | R | 0.38 | 3.39 | 0.03 |
|  |  | Q | 0.16 | 1.01 | 0.42 |
|  | *Myodes rutilus* | D | 0.21 | 1.22 | 0.34 |
|  |  | R | 0.17 | 0.93 | 0.45 |
|  |  | Q | 0.23 | 1.33 | 0.30 |
|  | *Neomys fodiens* | D | 0.41 | 6.13 | 0.06 |
|  |  | R | 0.31 | 3.99 | 0.08 |
|  |  | Q | 0.28 | 3.47 | 0.10 |
|  | *Sorex araneus* | D | 0.53 | 3.32 | 0.10 |
|  |  | R | 0.60 | 4.51 | 0.04 |
|  |  | Q | 0.33 | 1.45 | 0.39 |
| Mites | *Apodemus agrarius* | D | 0.24 | 1.23 | 0.26 |
|  |  | R | 0.13 | 0.60 | 0.72 |
|  |  | Q | 0.27 | 1.49 | 0.26 |
|  | *Arvicola amphibius* | D | 0.78 | 10.65 | 0.03 |
|  |  | R | 0.70 | 6.88 | 0.01 |
|  |  | Q | 0.78 | 10.37 | 0.03 |
|  | *Craseomys rufocanus* | D | 0.39 | 2.53 | 0.13 |
|  |  | R | 0.35 | 2.18 | 0.20 |
|  |  | Q | 0.38 | 2.48 | 0.13 |
|  | *Microtus arvalis* | D | 0.00 | 0.00 | 0.98 |
|  |  | R | 0.00 | 0.00 | 0.93 |
|  |  | Q | 0.00 | 0.00 | 0.95 |
|  | *Microtus oeconomus* | D | 0.33 | 3.44 | 0.06 |
|  |  | R | 0.25 | 2.38 | 0.09 |
|  |  | Q | 0.32 | 3.22 | 0.07 |
|  | *Mus musculus* | D | 0.21 | 1.06 | 0.38 |
|  |  | R | 0.64 | 7.10 | 0.10 |
|  |  | Q | 0.00 | 0.00 | 0.99 |
|  | *Myodes glareolus* | D | 0.01 | 0.05 | 0.79 |
|  |  | R | 0.01 | 0.10 | 0.76 |
|  |  | Q | 0.13 | 1.36 | 0.28 |
|  | *Myodes rutilus* | D | 0.12 | 1.05 | 0.37 |
|  |  | R | 0.09 | 0.75 | 0.47 |
|  |  | Q | 0.10 | 0.84 | 0.46 |
|  | *Ondatra zibeticus* | D | 0.18 | 1.06 | 0.42 |
|  |  | R | 0.12 | 0.67 | 0.76 |
|  |  | Q | 0.21 | 1.30 | 0.21 |
|  | *Sorex araneus* | D | 0.07 | 0.55 | 0.58 |
|  |  | R | 0.13 | 1.05 | 0.36 |
|  |  | Q | 0.06 | 0.42 | 0.66 |

**Table S5.** Summary of distance-based multivariate analyses of variance (db-MANOVAs) testing for differences in the DRQ composition of flea or mite assemblages harboured by the same host species between continental sections. Np: number of populations of a host species for which flea or mite DRQ composition was calculated.

| Parasite | Host species | Np | Proportion of variance explained | *F* | *p* |
| --- | --- | --- | --- | --- | --- |
| Fleas | *Apodemus agrarius* | 12 | 0.56 | 5.73 | 0.01 |
|  | *Apodemus uralensis* | 18 | 0.37 | 4.42 | 0.03 |
|  | *Arvicola amphibius* | 13 | 0.32 | 1.43 | 0.28 |
|  | *Cricetulus migratorius* | 16 | 0.04 | 0.59 | 0.48 |
|  | *Microtus agrestis* | 8 | 0.04 | 0.23 | 0.87 |
|  | *Microtus arvalis* | 19 | 0.32 | 2.40 | 0.10 |
|  | *Microtus gregalis* | 6 | 0.23 | 1.21 | 0.38 |
|  | *Microtus oeconomus* | 8 | 0.02 | 0.12 | 0.63 |
|  | *Mus musculus* | 25 | 0.18 | 1.13 | 0.35 |
|  | *Myodes glareolus* | 13 | 0.21 | 1.31 | 0.28 |
|  | *Myodes rutilus* | 11 | 0.06 | 0.28 | 0.83 |
|  | *Neomys fodiens* | 7 | 0.42 | 3.60 | 0.10 |
|  | *Rattus norvegicus* | 6 | 0.22 | 1.14 | 0.39 |
|  | *Sorex araneus* | 11 | 0.05 | 0.48 | 0.52 |
| Mites | *Craseomys rufocanus* | 8 | 0.09 | 0.59 | 0.51 |
|  | *Microtus arvalis* | 9 | 0.03 | 0.18 | 0.75 |
|  | *Microtus oeconomus* | 15 | 0.37 | 3.52 | 0.06 |
|  | *Myodes glareolus* | 8 | 0.26 | 2.13 | 0.22 |
|  | *Myodes rutilus* | 18 | 0.04 | 0.20 | 0.97 |
|  | *Sorex araneus* | 14 | 0.40 | 3.61 | 0.07 |

**Table S6.** Continental scale. Summary of permutational univariate ANOVAs testing for differences in the DRQ values (D: Simpson’s dominance; Q: Rao’s quadratic diversity; R: functional redundancy) of flea and mite assemblages harboured by the same host species between continental sections.

| Parasite | Host species | D/R/Q | *R^2^* | *F* | *p* |
| --- | --- | --- | --- | --- | --- |
| Fleas | Apodemus agrarius | D | 0.57 | 5.93 | 0.01 |
|  |  | R | 0.70 | 10.70 | 0.01 |
|  |  | Q | 0.29 | 1.85 | 0.20 |
|  | *Apodemus uralensis* | D | 0.37 | 4.42 | 0.03 |
|  |  | R | 0.59 | 10.86 | 0.00 |
|  |  | Q | 0.16 | 1.43 | 0.27 |
|  | *Arvicola amphibius* | D | 0.32 | 1.43 | 0.29 |
|  |  | R | 0.38 | 1.84 | 0.21 |
|  |  | Q | 0.17 | 0.62 | 0.63 |
|  | *Cricetulus migratorius* | D | 0.03 | 0.41 | 0.54 |
|  |  | R | 0.00 | 0.06 | 0.79 |
|  |  | Q | 0.08 | 1.25 | 0.30 |
|  | *Microtus agrestis* | D | 0.03 | 0.20 | 0.89 |
|  |  | R | 0.00 | 0.00 | 0.91 |
|  |  | Q | 0.11 | 0.74 | 0.60 |
|  | *Microtus arvalis* | D | 0.31 | 2.30 | 0.12 |
|  |  | R | 0.52 | 5.46 | 0.01 |
|  |  | Q | 0.18 | 1.11 | 0.38 |
|  | *Microtus gregalis* | D | 0.26 | 1.37 | 0.37 |
|  |  | R | 0.01 | 0.03 | 0.89 |
|  |  | Q | 0.40 | 2.68 | 0.10 |
|  | *Microtus oeconomus* | D | 0.02 | 0.10 | 0.65 |
|  |  | R | 0.00 | 0.02 | 0.94 |
|  |  | Q | 0.02 | 0.14 | 0.63 |
|  | *Mus musculus* | D | 0.19 | 1.15 | 0.36 |
|  |  | R | 0.24 | 1.58 | 0.21 |
|  |  | Q | 0.15 | 0.91 | 0.47 |
|  | *Myodes glareolus* | D | 0.16 | 0.94 | 0.42 |
|  |  | R | 0.11 | 0.64 | 0.55 |
|  |  | Q | 0.46 | 4.30 | 0.04 |
|  | *Myodes rutilus* | D | 0.06 | 0.25 | 0.82 |
|  |  | R | 0.17 | 0.81 | 0.47 |
|  |  | Q | 0.02 | 0.10 | 0.93 |
|  | *Neomys fodiens* | D | 0.39 | 3.16 | 0.16 |
|  |  | R | 0.72 | 13.17 | 0.01 |
|  |  | Q | 0.01 | 0.04 | 0.76 |
|  | *Rattus norvegicus* | D | 0.22 | 1.14 | 0.32 |
|  |  | R | 0.29 | 1.67 | 0.36 |
|  |  | Q | 0.18 | 0.85 | 0.32 |
|  | *Sorex araneus* | D | 0.04 | 0.37 | 0.55 |
|  |  | R | 0.17 | 1.88 | 0.19 |
|  |  | Q | 0.00 | 0.01 | 0.93 |
| Mites | *Craseomys rufocanus* | D | 0.09 | 0.61 | 0.48 |
|  |  | R | 0.09 | 0.61 | 0.47 |
|  |  | Q | 0.09 | 0.56 | 0.50 |
|  | *Microtus arvalis* | D | 0.02 | 0.18 | 0.67 |
|  |  | R | 0.01 | 0.08 | 0.78 |
|  |  | Q | 0.03 | 0.19 | 0.66 |
|  | *Microtus oeconomus* | D | 0.39 | 2.35 | 0.14 |
|  |  | R | 0.35 | 1.99 | 0.17 |
|  |  | Q | 0.35 | 1.99 | 0.18 |
|  | *Myodes glareolus* | D | 0.34 | 3.14 | 0.13 |
|  |  | R | 0.27 | 2.20 | 0.23 |
|  |  | Q | 0.08 | 0.51 | 0.54 |
|  | *Myodes rutilus* | D | 0.07 | 0.36 | 0.78 |
|  |  | R | 0.05 | 0.22 | 0.88 |
|  |  | Q | 0.04 | 0.20 | 0.89 |
|  | *Sorex araneus* | D | 0.40 | 3.61 | 0.07 |
|  |  | R | 0.50 | 5.43 | 0.02 |
|  |  | Q | 0.35 | 3.00 | 0.10 |

**Table S7.** Continental scale. Summary of permutational univariate ANOVAs testing for differences in the DRQ values (D: Simpson’s dominance; Q: Rao’s quadratic diversity; R: functional redundancy) of flea and mite assemblages between host species within a biome.

| Parasite | Biome | D/R/Q | *R^2^* | *F* | *p* |
| --- | --- | --- | --- | --- | --- |
| Fleas | Boreal forests | D | 0.55 | 4.70 | <0.001 |
|  |  | R | 0.44 | 2.97 | 0.01 |
|  |  | Q | 0.64 | 6.84 | <0.001 |
|  | Deserts | D | 0.09 | 0.32 | 0.89 |
|  |  | R | 0.17 | 0.65 | 0.66 |
|  |  | Q | 0.16 | 0.62 | 0.69 |
|  | Mountains | D | 0.38 | 2.28 | 0.01 |
|  |  | R | 0.24 | 1.17 | 0.31 |
|  |  | Q | 0.45 | 3.09 | <0.001 |
|  | Steppes | D | 0.41 | 2.02 | 0.04 |
|  |  | R | 0.38 | 1.81 | 0.08 |
|  |  | Q | 0.43 | 2.19 | 0.03 |
|  | Temperate forests | D | 0.65 | 5.87 | <0.001 |
|  |  | R | 0.51 | 3.36 | <0.001 |
|  |  | Q | 0.66 | 6.34 | 0.00 |
| Mites | Boreal forests | D | 0.53 | 5.09 | <0.001 |
|  |  | R | 0.38 | 2.68 | <0.001 |
|  |  | Q | 0.53 | 4.92 | <0.001 |
|  | Steppes | D | 0.65 | 5.49 | 0.01 |
|  |  | R | 0.66 | 5.88 | <0.001 |
|  |  | Q | 0.59 | 4.29 | 0.01 |
|  | Temperate forests | D | 0.17 | 0.63 | 0.69 |
|  |  | R | 0.33 | 1.47 | 0.26 |
|  |  | Q | 0.12 | 0.42 | 0.83 |

**Table S8.** Continental scale. Summary of permutational univariate ANOVAs testing for differences in the DRQ values (D: Simpson’s dominance; Q: Rao’s quadratic diversity; R: functional redundancy) of flea and mite assemblages between host species within a continental section.

| Parasite | Continental section | D/R/Q | *R^2^* | *F* | *p* |
| --- | --- | --- | --- | --- | --- |
| Fleas | Asian Far East | D | 0.80 | 12.55 | <0.001 |
|  |  | R | 0.73 | 8.87 | <0.001 |
|  |  | Q | 0.77 | 10.91 | <0.001 |
|  | Caucasus | D | 0.48 | 2.75 | 0.04 |
|  |  | R | 0.31 | 1.32 | 0.30 |
|  |  | Q | 0.59 | 4.26 | 0.01 |
|  | Central Asia | D | 0.27 | 1.45 | 0.13 |
|  |  | R | 0.19 | 0.93 | 0.54 |
|  |  | Q | 0.33 | 1.92 | 0.03 |
|  | Europe | D | 0.40 | 2.75 | 0.01 |
|  |  | R | 0.47 | 3.62 | <0.001 |
|  |  | Q | 0.38 | 2.59 | 0.01 |
|  | Southern Siberia | D | 0.18 | 0.58 | 0.63 |
|  |  | R | 0.25 | 0.90 | 0.48 |
|  |  | Q | 0.25 | 0.88 | 0.50 |
|  | Western Siberia | D | 0.34 | 1.43 | 0.17 |
|  |  | R | 0.45 | 2.26 | 0.02 |
|  |  | Q | 0.48 | 2.60 | 0.01 |
| Mites | Asian Far East | D | 0.03 | 0.13 | 0.69 |
|  |  | R | 0.10 | 0.42 | 0.95 |
|  |  | Q | 0.06 | 0.27 | 0.65 |
|  | Europe | D | 0.47 | 3.59 | 0.03 |
|  |  | R | 0.52 | 4.27 | 0.01 |
|  |  | Q | 0.46 | 3.36 | 0.03 |
|  | Northern Siberia | D | 0.05 | 0.23 | 0.78 |
|  |  | R | 0.05 | 0.23 | 0.82 |
|  |  | Q | 0.25 | 1.52 | 0.26 |
|  | Southern Siberia | D | 0.31 | 1.82 | 0.20 |
|  |  | R | 0.32 | 1.86 | 0.16 |
|  |  | Q | 0.22 | 1.10 | 0.39 |
|  | Western Siberia | D | 0.61 | 6.23 | <0.001 |
|  |  | R | 0.48 | 3.63 | <0.001 |
|  |  | Q | 0.56 | 5.06 | <0.001 |

**Table S9.** Regional scale. Summary of permutational univariate ANOVAs testing for differences in the DRQ values (D: Simpson’s dominance; Q: Rao’s quadratic diversity; R: functional redundancy) of flea and mite assemblages harboured by the same host species in different habitat types.

| Parasite | Host species | D/R/Q | *R^2^* | *F* | *p* |
| --- | --- | --- | --- | --- | --- |
| Fleas | *Apodemus agrarius* | D | 0.05 | 0.86 | 0.51 |
|  |  | R | 0.08 | 1.36 | 0.24 |
|  |  | Q | 0.12 | 1.93 | 0.09 |
|  | *Apodemus flavicollis* | D | 0.06 | 1.41 | 0.22 |
|  |  | R | 0.10 | 2.37 | 0.06 |
|  |  | Q | 0.12 | 2.85 | 0.01 |
|  | *Apodemus uralensis* | D | 0.06 | 0.63 | 0.60 |
|  |  | R | 0.18 | 2.27 | 0.09 |
|  |  | Q | 0.00 | 0.05 | 0.98 |
|  | *Microtus arvalis* | D | 0.06 | 0.63 | 0.60 |
|  |  | R | 0.18 | 2.27 | 0.09 |
|  |  | Q | 0.00 | 0.05 | 0.98 |
|  | *Myodes glareolus* | D | 0.23 | 4.37 | <0.001 |
|  |  | R | 0.25 | 4.96 | <0.001 |
|  |  | Q | 0.10 | 1.54 | 0.19 |
| Mites | *Apodemus agrarius* | D | 0.04 | 0.55 | 0.73 |
|  |  | R | 0.03 | 0.40 | 0.85 |
|  |  | Q | 0.05 | 0.68 | 0.63 |
|  | *Apodemus flavicollis* | D | 0.12 | 2.62 | 0.03 |
|  |  | R | 0.11 | 2.47 | 0.03 |
|  |  | Q | 0.11 | 2.58 | 0.03 |
|  | *Apodemus uralensis* | D | 0.04 | 0.51 | 0.59 |
|  |  | R | 0.02 | 0.30 | 0.74 |
|  |  | Q | 0.12 | 1.69 | 0.19 |
|  | *Microtus arvalis* | D | 0.03 | 0.17 | 0.91 |
|  |  | R | 0.07 | 0.46 | 0.70 |
|  |  | Q | 0.02 | 0.12 | 0.94 |
|  | *Microtus subterraneus* | D | 0.03 | 0.21 | 0.81 |
|  |  | R | 0.23 | 2.09 | 0.15 |
|  |  | Q | 0.06 | 0.45 | 0.64 |
|  | *Myodes glareolus* | D | 0.23 | 1.92 | 0.15 |
|  |  | R | 0.13 | 0.97 | 0.42 |
|  |  | Q | 0.28 | 2.45 | 0.09 |

**Table S10.** Regional scale. Summary of permutational univariate ANOVAs testing for differences in the DRQ values (D: Simpson’s dominance; Q: Rao’s quadratic diversity; R: functional redundancy) of flea and mite assemblages harboured by the same host species in different localities.

| Parasite | Host species | D/R/Q | *R^2^* | *F* | *p* |
| --- | --- | --- | --- | --- | --- |
| Fleas | *Apodemus agrarius* | D | 0.13 | 2.31 | 0.04 |
|  |  | R | 0.08 | 1.23 | 0.29 |
|  |  | Q | 0.18 | 3.23 | 0.01 |
|  | *Apodemus flavicollis* | D | 0.21 | 3.82 | 0.00 |
|  |  | R | 0.23 | 4.32 | 0.00 |
|  |  | Q | 0.36 | 7.84 | 0.00 |
|  | *Apodemus uralensis* | D | 0.18 | 3.61 | 0.03 |
|  |  | R | 0.22 | 4.71 | 0.00 |
|  |  | Q | 0.05 | 0.89 | 0.42 |
|  | *Microtus arvalis* | D | 0.01 | 0.14 | 0.71 |
|  |  | R | 0.01 | 0.18 | 0.66 |
|  |  | Q | 0.00 | 0.03 | 0.91 |
|  | *Myodes glareolus* | D | 0.30 | 4.57 | 0.00 |
|  |  | R | 0.30 | 4.48 | 0.00 |
|  |  | Q | 0.35 | 5.55 | 0.00 |
| Mites | *Apodemus agrarius* | D | 0.06 | 1.04 | 0.39 |
|  |  | R | 0.05 | 0.78 | 0.55 |
|  |  | Q | 0.07 | 1.22 | 0.31 |
|  | *Apodemus flavicollis* | D | 0.14 | 2.54 | 0.02 |
|  |  | R | 0.14 | 2.67 | 0.02 |
|  |  | Q | 0.12 | 2.28 | 0.04 |
|  | *Apodemus uralensis* | D | 0.15 | 2.21 | 0.10 |
|  |  | R | 0.26 | 4.50 | 0.02 |
|  |  | Q | 0.04 | 0.54 | 0.60 |
|  | *Microtus arvalis* | D | 0.10 | 0.71 | 0.54 |
|  |  | R | 0.11 | 0.85 | 0.47 |
|  |  | Q | 0.09 | 0.67 | 0.58 |
|  | *Microtus subterraneus* | D | 0.01 | 0.04 | 0.96 |
|  |  | R | 0.09 | 0.38 | 0.68 |
|  |  | Q | 0.01 | 0.04 | 0.94 |
|  | *Myodes glareolus* | D | 0.06 | 0.35 | 0.78 |
|  |  | R | 0.03 | 0.16 | 0.92 |
|  |  | Q | 0.13 | 0.81 | 0.49 |

**Table S11.** Regional scale. Summary of permutational univariate ANOVAs testing for differences in the DRQ values (D: Simpson’s dominance; Q: Rao’s quadratic diversity; R: functional redundancy) of flea and mite assemblages harboured by different host species within a habitat type.

| Parasite | Habitat type | D/R/Q | *R^2^* | *F* | *p* |
| --- | --- | --- | --- | --- | --- |
| Fleas | Lowland fields | D | 0.20 | 5.19 | 0.01 |
|  |  | R | 0.01 | 0.26 | 0.77 |
|  |  | Q | 0.23 | 6.06 | 0.01 |
|  | Lowland forests | D | 0.00 | 0.05 | 0.95 |
|  |  | R | 0.19 | 2.96 | 0.06 |
|  |  | Q | 0.04 | 0.46 | 0.63 |
|  | Lowland river valleys | D | 0.08 | 0.84 | 0.48 |
|  |  | R | 0.07 | 0.64 | 0.58 |
|  |  | Q | 0.12 | 1.25 | 0.30 |
|  | Lowland woodland belts | D | 0.06 | 1.37 | 0.24 |
|  |  | R | 0.16 | 3.78 | 0.01 |
|  |  | Q | 0.08 | 1.72 | 0.14 |
|  | Montane forests | D | 0.13 | 2.92 | 0.06 |
|  |  | R | 0.21 | 4.93 | 0.01 |
|  |  | Q | 0.03 | 0.59 | 0.55 |
|  | Montane river valleys | D | 0.16 | 3.30 | 0.01 |
|  |  | R | 0.16 | 3.43 | 0.01 |
|  |  | Q | 0.11 | 2.14 | 0.08 |
|  | Montane shrubbery | D | 0.30 | 5.46 | 0.02 |
|  |  | R | 0.37 | 7.47 | 0.01 |
|  |  | Q | 0.21 | 3.56 | 0.06 |
| Mites | Lowland fields | D | 0.10 | 1.49 | 0.24 |
|  |  | R | 0.00 | 0.03 | 0.96 |
|  |  | Q | 0.26 | 4.86 | 0.01 |
|  | Lowland forests | D | 0.52 | 24.04 | <0.001 |
|  |  | R | 0.61 | 35.01 | <0.001 |
|  |  | Q | 0.42 | 15.99 | <0.001 |
|  | Lowland river valleys | D | 0.51 | 8.71 | <0.001 |
|  |  | R | 0.57 | 10.85 | <0.001 |
|  |  | Q | 0.44 | 6.41 | <0.001 |
|  | Lowland woodland belts | D | 0.36 | 6.54 | <0.001 |
|  |  | R | 0.39 | 7.48 | <0.001 |
|  |  | Q | 0.34 | 6.01 | <0.001 |
|  | Montane river valleys | D | 0.27 | 4.72 | <0.001 |
|  |  | R | 0.38 | 7.76 | <0.001 |
|  |  | Q | 0.15 | 2.22 | 0.07 |
|  | Montane shrubbery | D | 0.19 | 3.84 | 0.03 |
|  |  | R | 0.27 | 5.89 | 0.01 |
|  |  | Q | 0.16 | 2.96 | 0.06 |

**Table S12.** Regional scale. Summary of permutational univariate ANOVAs testing for differences in the DRQ values (D: Simpson’s dominance; Q: Rao’s quadratic diversity; R: functional redundancy) of flea and mite assemblages harboured by different host species within a locality.

| Parasite | Locality | D/R/Q | *R^2^* | *F* | *p* |
| --- | --- | --- | --- | --- | --- |
| Fleas | Hran | D | 0.08 | 2.41 | 0.04 |
|  |  | R | 0.03 | 0.84 | 0.50 |
|  |  | Q | 0.13 | 4.39 | <0.001 |
|  | Hylov | D | 0.33 | 5.20 | 0.01 |
|  |  | R | 0.32 | 4.88 | 0.02 |
|  |  | Q | 0.30 | 4.54 | 0.02 |
|  | Javorie | D | 0.17 | 3.39 | 0.02 |
|  |  | R | 0.29 | 6.64 | <0.001 |
|  |  | Q | 0.19 | 3.79 | 0.01 |
|  | Kavecany | D | 0.19 | 1.75 | 0.20 |
|  |  | R | 0.02 | 0.16 | 0.85 |
|  |  | Q | 0.27 | 2.75 | 0.09 |
|  | Kosice | D | 0.12 | 2.05 | 0.14 |
|  |  | R | 0.11 | 1.82 | 0.18 |
|  |  | Q | 0.11 | 1.96 | 0.15 |
|  | Kosicka Kotlina | D | 0.34 | 4.37 | 0.01 |
|  |  | R | 0.22 | 2.42 | 0.09 |
|  |  | Q | 0.30 | 3.64 | 0.02 |
|  | Slovensky Kras | D | 0.87 | 26.97 | 0.10 |
|  |  | R | 0.03 | 0.12 | 0.58 |
|  |  | Q | 0.93 | 53.27 | 0.09 |
|  | Svata Maria | D | 0.17 | 1.58 | 0.21 |
|  |  | R | 0.10 | 0.82 | 0.49 |
|  |  | Q | 0.27 | 2.86 | 0.05 |
| Mites | Hran | D | 0.49 | 22.72 | <0.001 |
|  |  | R | 0.52 | 25.35 | <0.001 |
|  |  | Q | 0.42 | 17.39 | <0.001 |
|  | Hylov | D | 0.65 | 26.50 | <0.001 |
|  |  | R | 0.73 | 38.05 | <0.001 |
|  |  | Q | 0.56 | 18.15 | <0.001 |
|  | Javorie | D | 0.29 | 3.89 | 0.01 |
|  |  | R | 0.47 | 8.44 | <0.001 |
|  |  | Q | 0.12 | 1.34 | 0.26 |
|  | Kavecany | D | 0.13 | 0.80 | 0.46 |
|  |  | R | 0.29 | 2.25 | 0.15 |
|  |  | Q | 0.03 | 0.18 | 0.84 |
|  | Kosice | D | 0.41 | 5.04 | <0.001 |
|  |  | R | 0.53 | 8.18 | <0.001 |
|  |  | Q | 0.33 | 3.55 | 0.02 |
|  | Kosicka Kotlina | D | 0.14 | 1.04 | 0.40 |
|  |  | R | 0.27 | 2.26 | 0.09 |
|  |  | Q | 0.10 | 0.71 | 0.58 |
|  | Svata Maria | D | 0.29 | 4.44 | 0.02 |
|  |  | R | 0.33 | 5.31 | 0.01 |
|  |  | Q | 0.24 | 3.46 | 0.04 |
